# Severe nodulocystic acne requiring corticosteroids after social media-induced delay: a two-patient case series

**DOI:** 10.1093/skinhd/vzaf074

**Published:** 2025-10-09

**Authors:** Nicolò Rivetti, Valeria Brazzelli, Carlo Francesco Tomasini, Stefania Barruscotti

**Affiliations:** Dermatology Outpatient Clinic, Istituto Clinico Beato Matteo, Gruppo San Donato, Vigevano, Italy; Dermatologic Clinic, Fondazione IRCCS Policlinico San Matteo, Pavia, Italy; Department of Clinical, Surgical, Diagnostic and Pediatric Sciences, Institute of Dermatology, Università degli Studi di Pavia, Pavia, Italy; Dermatologic Clinic, Fondazione IRCCS Policlinico San Matteo, Pavia, Italy; Department of Clinical, Surgical, Diagnostic and Pediatric Sciences, Institute of Dermatology, Università degli Studi di Pavia, Pavia, Italy; Dermatologic Clinic, Fondazione IRCCS Policlinico San Matteo, Pavia, Italy; Department of Clinical, Surgical, Diagnostic and Pediatric Sciences, Institute of Dermatology, Università degli Studi di Pavia, Pavia,Italy

## Abstract

Social media platforms, particularly TikTok, are increasingly used by adolescents for skincare advice. However, much of the content lacks medical accuracy and may delay appropriate treatment. We report two adolescent boys with severe nodulocystic acne who postponed dermatology care while following influencer-promoted routines. In both cases, the disease progressed to a highly inflammatory state, prompting the use of systemic corticosteroids before starting isotretinoin. The first patient, aged 16 years, presented with painful nodules after months of self-treatment; a short course of oral prednisone and antibiotics was followed by isotretinoin, with a good response. The second patient, aged 15 years, experienced similar progression and was also pretreated with oral steroids before isotretinoin initiation. Both achieved clinical improvement. These cases highlight the therapeutic consequences of digital misinformation and the role of corticosteroids as effective bridging therapy. They underscore the importance of early dermatology intervention and the need for evidence-based communication in online health spaces.

## Teaser

Delayed by TikTok advice, two adolescents with nodulocystic acne required systemic steroids before isotretinoin, underscoring a modern challenge in acne management.

What is already known about this topic?

- Social media platforms, particularly TikTok, often spread unverified acne advice, leading many adolescents to delay dermatology consultation.
- Such delays can worsen inflammatory acne, increasing the risk of scarring and sometimes requiring corticosteroid bridging before isotretinoin.

What does this study add?

- This case series illustrates how social media-induced therapeutic delay can escalate nodulocystic acne to a state requiring systemic corticosteroids before isotretinoin initiation.
- It emphasizes the clinical impact of digital misinformation and highlights corticosteroid bridging as a practical option in severe, delayed-treatment cases.

Dermatology has entered the algorithmic age: on platforms like TikTok, skincare advice spreads faster than clinical guidelines. Social media, particularly TikTok, has become the leading source of skincare information for adolescents, often prioritizing virality over medical accuracy. Recent studies estimate that over 70% of teenagers consult TikTok for acne advice before seeing a physician.^1–3^ TikTok, in particular, hosts thousands of short videos on skincare, many of which promote over-the-counter routines for acne management. Although these videos are visually engaging and widely shared, they often lack medical accuracy and may lead to self-treatment based on incomplete or incorrect advice.^1–3^ This phenomenon can be especially problematic in cases of inflammatory or nodulocystic acne, where timely medical intervention is essential to prevent scarring and psychological distress.^4^

We report two cases of adolescent boys who delayed dermatological consultation due to misplaced confidence in social media-based skincare routines. In both cases, the acne progressed to a highly inflammatory stage requiring systemic corticosteroids before isotretinoin could be safely initiated. These cases underscore the clinical and therapeutic consequences of digital misinformation and highlight the importance of early dermatology evaluation.

## Case report

### Patient 1

**Figure 1:**
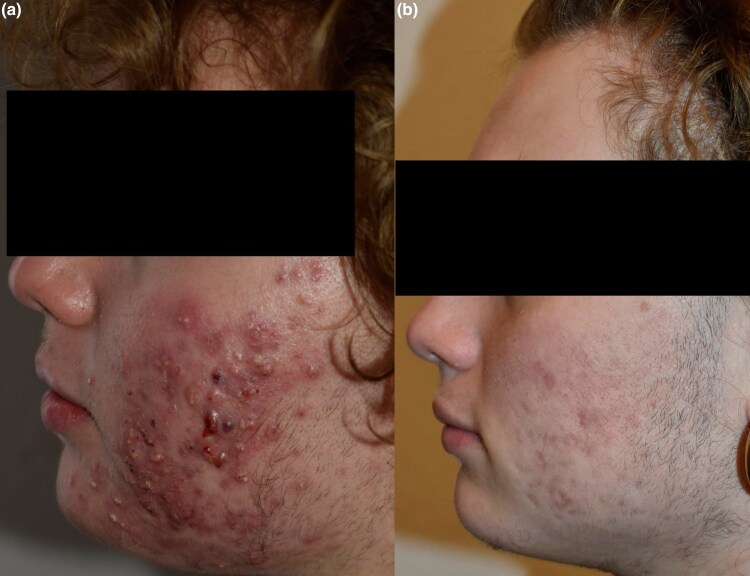
(a) Initial presentation of the first patient, a 16-year-old boy with painful, severely inflamed nodulocystic acne affecting the face and trunk. Several lesions show early signs of scarring. (b) Clinical appearance after 3 months of treatment with oral isotretinoin following a short course of prednisone and clarithromycin. Inflammatory activity is markedly reduced, with no new nodules.

A 16-year-old boy presented in January 2025 with painful, severely inflamed nodulocystic acne involving the face, shoulders and upper back (Figure 1a). For several months, the patient and his mother had relied on dermocosmetic regimens promoted by TikTok influencers, including salicylic acid cleansers, niacinamide serums and clay masks, without medical supervision. The condition progressively worsened, and several lesions had begun to scar.

Upon dermatology evaluation, systemic treatment was deemed necessary. Due to the high level of inflammation and patient discomfort, oral prednisone was initiated at 0.5 mg kg^–1^ daily for 7 days, along with clarithromycin 500 mg daily for 10 days. After the acute flare subsided, oral isotretinoin was introduced at 0.3 mg kg^–1^ daily and gradually increased. Over the following 3 months, the patient showed marked improvement with no new nodules and resolution of active inflammation (Figure 1b).

### Patient 2

**Figure 2:**
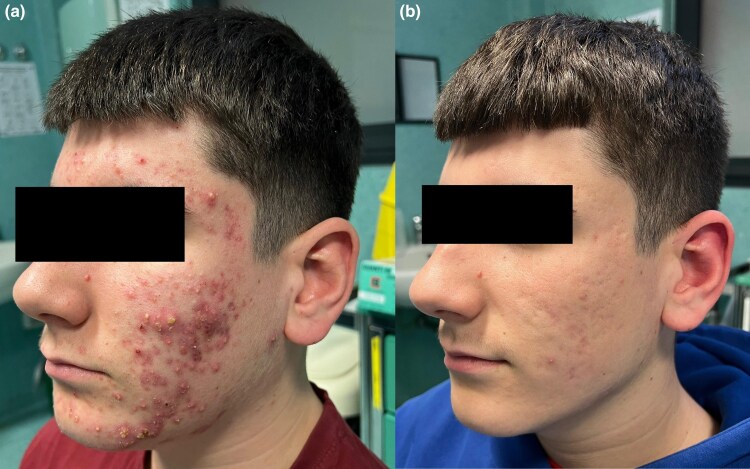
(a) Presentation of the second patient, a 15-year-old boy with nodulocystic acne primarily affecting the face and chest. The patient had delayed medical consultation after relying on social media–based skincare routines. (b) Clinical improvement after 10 weeks of treatment with oral isotretinoin, initiated after a short bridging course of systemic corticosteroids.

A 15-year-old boy presented in February 2025 with similar features of nodulocystic acne (Figure 2a), primarily affecting the face and chest. The patient reported having followed viral ‘skinfluencer’ routines for over 3 months, including oil-free moisturizers, exfoliating toners and benzoyl peroxide–based gels, all without professional input. His family delayed medical consultation, believing that ‘natural’ approaches would eventually work.

The acne had progressed to a painful, inflamed state with early signs of scarring. As with the first patient, a short course of oral prednisone (0.5 mg kg^–1^ daily for 5 days) was prescribed to reduce inflammatory activity. Isotretinoin was then initiated and titrated gradually. By ­follow-up at 10 weeks, there was significant improvement in both inflammatory and nodular lesions (Figure 2b), with no further scarring.

To our knowledge, this is among the few clinical reports to document corticosteroid bridging in nodulocystic acne as a direct consequence of social media–induced therapeutic delay.

## Discussion

These two cases illustrate the potential clinical consequences of social media misinformation in dermatology. Both adolescents experienced a delay in appropriate medical treatment for severe acne due to reliance on skincare advice circulated via TikTok.^1–4^ The resulting disease progression necessitated systemic intervention, including the use of oral corticosteroids prior to standard isotretinoin therapy. Such delays can escalate an otherwise manageable condition into one requiring more aggressive and risk-prone systemic therapy, thereby increasing the risk of complications such as scarring and isotretinoin flare.^5,6^

While oral isotretinoin remains the gold standard for severe nodulocystic acne,^5,6^ it is well known that initiating it in the context of high inflammatory burden may lead to worsening, or ‘flare’ phenomena. In these situations, systemic corticosteroids – particularly short courses of oral prednisone – are recognized as effective bridging strategies to control inflammation and improve patient comfort, especially in cases with deep, painful nodules and risk of scarring.^6–8^ This therapeutic approach is mentioned in several clinical guidelines and has a well-established role in acne management, despite not being routinely required for all patients.^5,6,8^ The additional psychosocial burden observed in these cases reflects a broader issue: adolescents increasingly consult nonmedical digital sources before seeking medical care.^1–3^ The appeal of ‘quick fix’ skincare routines and distrust in pharmacologic treatments may delay necessary intervention and worsen long-term outcomes. In both cases, parental hesitation further contributed to the delay, a common factor when systemic medications like isotretinoin or corticosteroids are proposed.^9^

These cases reinforce the importance of early dermatologic evaluation and evidence-based treatment, particularly for patients with aggressive inflammatory phenotypes. Moreover, they call attention to the need for dermatologists to engage more actively in public communication, particularly on digital platforms where misinformation often circulates unchecked.^1,4^ Given the volume and reach of such misinformation, it becomes essential for dermatologists to be not only reactive in clinical settings, but also proactive in reclaiming digital space through collaboration with credible content creators, dermatology societies or patient advocacy platforms. Finally, in adolescents with rapidly progressive nodulocystic acne and reluctance toward systemic therapy, short-course corticosteroids can serve not only to reduce inflammation but also to ease parental concern and improve adherence to isotretinoin.

## Data Availability

No datasets were generated or analysed during the preparation of this case series.

## References

[1] Yousaf A, Hagen R, Delaney E et al The influence of social media on acne treatment: a cross-sectional survey. Pediatr Dermatol 2020; 37:301–4. 31944359 10.1111/pde.14091 PMC7453954

[2] Ertekin SS, Salici NS, Manav Bas V et al Influence of social media and internet on treatment decisions in adult female acne patients: a cross-sectional survey study. Dermatol Pract Concept 2024; 14:e2024156. 39122512 10.5826/dpc.1403a156 PMC11314130

[3] Finan CF, Leon P, Midani L et al Social media use among adolescents with acne: a cross-sectional survey study. Pediatr Dermatol 2024; 41:835–7. 38725265 10.1111/pde.15651

[4] Bal ZI, Karaosmanoglu N, Temel B et al Trust in dermatologists versus social media influencers among acne patients. Cureus 2025; 17:e83930. 40357320 10.7759/cureus.83930 PMC12068905

[5] Zaenglein AL, Pathy AL, Schlosser BJ et al Guidelines of care for the management of acne vulgaris. J Am Acad Dermatol 2016; 74:945–73. 26897386 10.1016/j.jaad.2015.12.037

[6] Gollnick H, Cunliffe W, Berson D et al Management of acne: a report from a Global Alliance to Improve Outcomes in Acne. J Am Acad Dermatol 2003; 49:S1–37. 12833004 10.1067/mjd.2003.618

[7] Cantrell W, Easley L, Squittieri K. Steroids used to treat acne vulgaris: a review of efficacy, safety, and clinical considerations. J Drugs Dermatol 2024; 23:404–9. 38834219 10.36849/JDD.7846

[8] Vasicek B, Adams W, Steadman L et al Coprescription of isotretinoin and systemic corticosteroids for acne: an analysis of the national ambulatory medical care survey. J Clin Aesthet Dermatol 2019; 12:27–8. PMC6624007 31360285

[9] Kara Polat A, Akin Belli A, Ergun EZ et al Knowledge ­levels and concerns about oral isotretinoin treatment in the parents of adolescent acne patients. Dermatol Ther 2020; 33:e13669. 32459383 10.1111/dth.13669

